# The Effect of Malicious Envy on Schadenfreude When Schadenfreude Is Elicited Through Social Comparisons

**DOI:** 10.3389/fpsyg.2021.769826

**Published:** 2021-12-13

**Authors:** Huiyan Lin, Jiafeng Liang

**Affiliations:** ^1^Institute of Applied Psychology, School of Public Administration, Guangdong University of Finance, Guangzhou, China; ^2^Laboratory for Behavioral and Regional Finance, Guangdong University of Finance, Guangzhou, China; ^3^School of Education, Guangdong University of Education, Guangzhou, China

**Keywords:** malicious envy, schadenfreude, social comparisons, gain, loss

## Abstract

Previous studies have investigated whether envy, particularly malicious envy, increases feelings of schadenfreude and whether this effect is evident in both gain and loss frames. However, as a social-comparison-based emotion, schadenfreude was not investigated through social comparisons in these previous studies. Thus, the present study aimed to investigate whether malicious envy influences schadenfreude when schadenfreude is elicited in the context of precise and ambiguous social comparisons. To address this issue, participants in the present study were asked to play a monetary game with several other players. In the experimental condition, participants gained less or lost more than the other player; in the control condition, both the participants and the player gained little or lost much. Subsequently, the participants observed that the player encountered a misfortune, that is, gained less or lost more money than the participant. The results showed that when participants knew the exact amount of monetary gained and lost by themselves and the other player (i.e., precise social comparisons), malicious envy increased feelings of schadenfreude only in the loss frame rather than in the gain frame. More importantly, malicious envy turned out to reduce feelings of schadenfreude in both gain and loss frames, when participants did not know the exact amount (i.e., ambiguous social comparisons). The findings provide novel evidence that malicious envy does not always increase schadenfreude particularly when schadenfreude is elicited through social comparisons.

## Introduction

Envy is a social-comparison-based emotion that is elicited when “a person lacks another’s superior quality, achievement, or possession and either desires it or wishes that the other lacked it” (Parrott and Smith, 1993). Envy is thought to be one of the most potent causes of unhappiness, and an envious person wishes to inflict misfortune on others (Russell, 1930). Therefore, when other individuals experience misfortune, it is often thought that the envious person will not sympathize with them and instead will feel malicious joy (i.e., schadenfreude; Smith, 2000). Nevertheless, it is of interest to understand whether envy increases schadenfreude in different situations.

Previous studies have often utilized a scenario task to investigate whether envy influences envious persons’ feelings of schadenfreude when misfortunes occur to enviable persons. Such tasks include two sequential parts (e.g., Feather and Sherman, 2002; Feather and Nairn, 2005; Van Dijk et al., 2006; Takahashi et al., 2009; Feather et al., 2013; Van de Ven et al., 2015; Baez et al., 2016, 2018; Santamaría-García et al., 2017; Lin and Liang, 2021). In the first part, participants encounter a person who is better than the participant in a specific domain (e.g., the person wins the lottery and the participant does not; that is, the experimental condition). In some cases, there is also a person who is similar to the participant in the related domain (e.g., both the person and the participant do not win the lottery). This manipulation is used as the control condition. In the second part, participants are told that the person they have just encountered experience an unfortunate event (e.g., the person does not win the subsequent lottery). The feelings of pleasure (schadenfreude) stimulated by others’ misfortune are assessed. The effects of envy on the feelings of schadenfreude are measured as either the correlation between envy and schadenfreude (when the control condition is not manipulated) or the differential feelings of schadenfreude elicited by the envy and control conditions (when the control condition is manipulated).

Using such tasks, a number of studies have shown that envy increases feelings of schadenfreude (e.g., Van Dijk et al., 2006; Takahashi et al., 2009; Cikara and Fiske, 2013; Feather et al., 2013; Baez et al., 2016, 2018; Santamaría-García et al., 2017). Our recent study further revealed that this effect was evident in both gain and loss frames (Lin and Liang, 2021). On neural levels, schadenfreude has been found to recruit a fronto-temporo-subcortical network (Shamay-Tsoory et al., 2007; Takahashi et al., 2009; Santamaría-García et al., 2017; Baez et al., 2020; for a review: Jankowski and Takahashi, 2014), such as reward-related striatal regions (Takahashi et al., 2009; Dvash et al., 2010; Baez et al., 2016, 2018, 2020; Santamaría-García et al., 2017; for a review: Jankowski and Takahashi, 2014). Increased schadenfreude in the influence of envy was thus reflected by altered fronto-temporo-subcortical network, increased activations of striatum in particular (Takahashi et al., 2009; Baez et al., 2016, 2018).

The effect of envy on schadenfreude might be explained by the achievements of the motivational goal of envy and/or reduced psychological pain. The motivational goal of malicious envy is to prevent another person from being better off. If the other person encounters misfortune, his or her superiority is reduced. In this case, the motivational goal is achieved, thus triggering positive feelings (i.e., schadenfreude; Van de Ven et al., 2015). Regarding psychological pain, envy is thought to be a pain emotion (Lange et al., 2018). Increased schadenfreude in the influence of envy is because other’s misfortune relieves individuals’ feelings of pain (Takahashi et al., 2009).

However, other studies did not replicate this effect of envy on schadenfreude. The studies have observed that schadenfreude is not caused by envy but by other factors, such as negative emotions (e.g., resentment, anger, and dislike, Feather and Sherman, 2002; Hareli and Weiner, 2002; Feather and Nairn, 2005), painful feelings of inferiority (Leach and Spears, 2008), and beliefs about morality (Brambilla and Riva, 2017). These findings might suggest that envy does not always increase feelings of schadenfreude.

Whether the effect of envy on schadenfreude appears have been suggested to be associated with several factors. First, previous studies have proposed two categories of envy, malicious, and benign envy (e.g., Van de Ven et al., 2009; Crusius and Mussweiler, 2012; Crusius and Lange, 2014; Falcon, 2015; Lange et al., 2016; Van de Ven, 2016; Briki, 2018; Vrabel et al., 2018; Xiang et al., 2018). Both of these categories of envy are negative emotions caused by an individual lacking something another person has. Malicious envy is associated with deservedness and is reduced by pulling the other person down, while benign envy is associated with feelings of control and is reduced by improving one’s own performance (Smith and Kim, 2007; Van de Ven et al., 2011a,b, 2012; Lange et al., 2016, 2018; Van de Ven, 2017). In studies that examined the two types of envy separately, schadenfreude was affected only by malicious envy and not by benign envy; more importantly, this effect was independent of other antecedents of schadenfreude (e.g., pain, inferiority, dislike, and anger; Van de Ven et al., 2015). Using a meta-analysis, Lange et al. (2018) also showed stronger and more positive connections between envy and schadenfreude when the relevant research operationalized malicious envy as distinct from pain or benign envy. In addition to different categories of envy, previous studies have also suggested that the effect of envy on schadenfreude is evident only when enviable persons are competitive out-group members (Cikara and Fiske, 2013) and when schadenfreude befalls an enviable person who is similar and might serve as a relevant social comparison (Van Dijk et al., 2006). Taken together, these findings might suggest that the effect of (malicious) envy on schadenfreude occurs only under certain circumstances.

Notably, social comparison theory suggests that a social-comparison-based emotion results from the implications of a comparison for the self (Smith, 2000). Both envy and schadenfreude are thought to be elicited by the process of social comparison (e.g., Smith, 2000; Ben-Ze'ev, 2001; Shamay-Tsoory et al., 2009). However, in previous studies, schadenfreude was not investigated in the context of social comparisons (i.e., the misfortune associated with schadenfreude was related only to others rather than to the differences between the participants and others; e.g., Feather and Sherman, 2002; Feather and Nairn, 2005; Van Dijk et al., 2006; Takahashi et al., 2009; Feather et al., 2013; Van de Ven et al., 2015; Baez et al., 2016, 2018; Santamaría-García et al., 2017; Lin and Liang, 2021). Importantly, if the misfortune involves social comparison, the assessment of schadenfreude can consider individuals’ evaluation of the relative inferiority or superiority of their own and another person’s attributes. This comparison may be integrated with preceding comparisons (e.g., those occurring during the elicitation of malicious envy), which might alter the effect of malicious envy on schadenfreude.

Furthermore, social comparisons are either precise (e.g., a person wins $100 more than someone else) or ambiguous (e.g., a person wins more than someone else, but the exact difference is unknown). During precise social comparisons, integrations between current and preceding outcomes for both individuals and others have individuals clearly affirm that their social status is not as inferior as it was used to be. This self-affirmation is thought to reduce schadenfreude (Van Dijk et al., 2011). Similarly, individuals during ambiguous social comparisons also know increased social status after outcome integrations, which might reduce feelings of schadenfreude. Moreover, due to ambiguous outcomes, individuals are uncertain about the extents social status increases (i.e., still inferior, similar, or superior). Previous studies have shown that uncertainty reduces pleasant feelings elicited by positive outcomes (Lin et al., 2012, 2015, 2020). The uncertainty about the variation of social status might also reduce schadenfreude.

Therefore, the present study aimed to investigate whether malicious envy influenced schadenfreude when both malicious envy and schadenfreude were elicited in the context of precise and ambiguous social comparisons. To address this issue, participants in the present study were required to play a monetary game similar to our previous study (Lin and Liang, 2021). The game included two rounds in each trial. The aim of the first round was to elicit a malicious envy emotion, and the aim of the second round was to assess schadenfreude. In the first round of the game, participants in the experimental (i.e., malicious envy) condition gained less money than the player in the win block and lost more in the loss block. For the control condition, both participants and players gained a little in the win block or lost a large amount in the loss block. In the second round, participants encountered a misfortune situation. As schadenfreude in this study was investigated through social comparisons, the situation was regarding the outcome was worse for the other players than for the participants (i.e., the players gained less money than the participants in the win block or lost more money in the loss block). The outcomes for participants and players and the outcome differences between them were precise in Experiment 1 but ambiguous in Experiment 2.

As mentioned above, malicious envy might not always increase schadenfreude when schadenfreude is elicited in the context of social comparisons (i.e., malicious envy might not influence schadenfreude in precise social comparisons and even reduce schadenfreude in ambiguous social comparisons), as individuals might affirm their increased social status after outcome integrations. Moreover, it has been shown that individuals in the gain frame are more sensitive to the differences between their own and the other’s outcomes than do those in the loss frame (De Dreu et al., 1992, 1994; Poppe and Valkenberg, 2003). The sensitivity in the gain frame might have participants be more likely to integrate current and preceding outcomes (e.g., both Round 1 and 2) between themselves and others and to realize their increased social status, resulting in reducing schadenfreude to a larger extent. Nevertheless, it is notable that the modulation of frame might be only evident in precise social comparisons, as in ambiguous social comparisons, schadenfreude might have been largely reduced by outcome uncertainty irrespective of frame. Taken together, we predict that malicious envy will increase schadenfreude only in the loss-associated precise social comparisons, whereas this will not be the case in gain-associated precise social comparisons or in gain- and loss-associated ambiguous comparisons.

## Experiment 1

### Methods

#### Participants

Thirty-five undergraduate students (ranging from 18 to 22 years old, *M* = 19.83, *SD* = 0.85; 18 females) were recruited as participants. Because our previous study revealed an effect of malicious envy on schadenfreude in gain and loss frames by using 32 participants (Lin and Liang, 2021), the sample size in the present study was likely sufficient. Participants reported normal or corrected-to-normal vision and no history of neurological illness. All participants gave written informed consent in accordance with the standard ethical guidelines defined in the Declaration of Helsinki. The study was approved by the local ethics committee.

#### Procedure

After informed consent was obtained and handedness was determined, the participant was seated in a comfortable chair in a quiet room approximately 100 cm directly in front of a 22-inch computer monitor with a screen resolution of 640 × 480 pixels. Stimulus presentation and behavioral data collection were controlled by E-Prime 2.0 software (Psychology Software Tools, Inc., Sharpsburg, PA, United States). All stimuli were presented against a dark background.

Prior to the actual experiment, each participant was told that he/she would play a monetary game with three anonymous players. Previous studies have suggested that the effect of envy, particularly malicious envy, on schadenfreude is evident when enviable persons are competitive out-group members (Cikara and Fiske, 2013) and when schadenfreude befalls an enviable person who is similar and might serve as a relevant social comparison (e.g., the enviable person is of the same sex as the envious person; Van Dijk et al., 2006). Therefore, in the present study, it was emphasized to the participants that the players were undergraduate students from other universities and were of the same sex as the participants themselves. The participants were informed that the players would play the game in other rooms; therefore, the participants and players could not see each other. In fact, there were no other players, and all the players’ choices in the experiment were predetermined by the experimental randomization. The participants were told that money could be won or lost based on 10% of the general tokens gained or lost across all trials with the addition or subtraction of a basic compensation of 10 RMB, respectively [e.g., if participants gained 10 tokens over all the trials in the game, then they would receive (10 + 10 × 10%) RMB; if they lost 10 tokens overall in the game, then they would receive (10–10 × 10%) RMB]. In fact, the general tokens gained or lost were randomized by a computer and ranged from −36 to +36.

**Figure 1:**
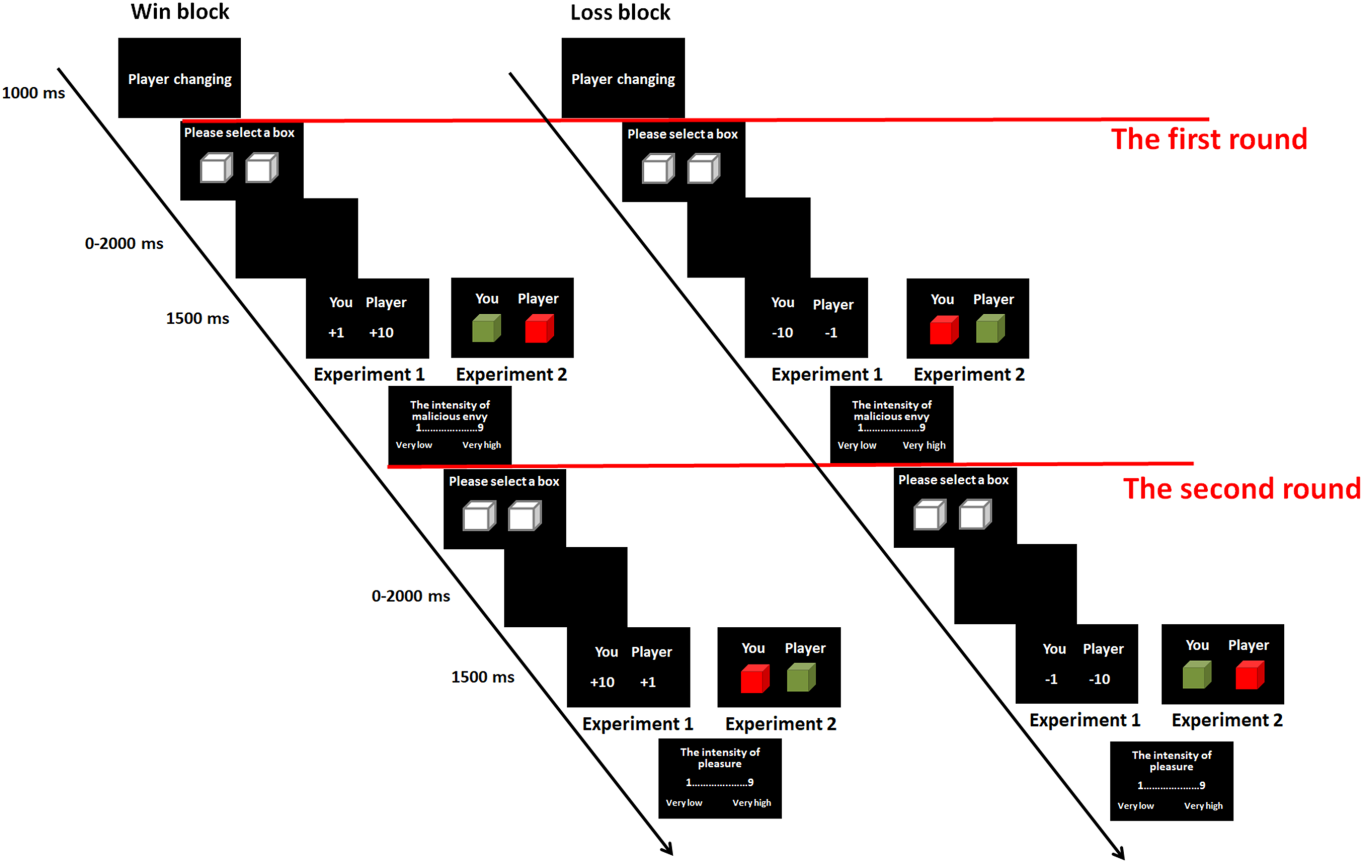
Experimental procedure in Experiment 1 and 2. Each trial includes two rounds. The first round was to elicit malicious envy, and the second round was to assess schadenfreude.

As illustrated in Figure 1, the actual experiment consisted of 2 blocks: win and loss blocks. The presentation sequence of the blocks was counterbalanced across participants. For both of the blocks, each trial started with the label “player changing” for 1000 ms. The label signified that the computers would select one of the three players in a randomized order for the next trial of the game. However, participants did not know which player would be chosen. Each trial consisted of two rounds. The aim of the first round was to elicit malicious envy, and the aim of the second round was to assess feelings of schadenfreude. In the first round, participants were presented with two white boxes, one to the left of the center of the screen and the other to the right. Participants were told that there was either 1 or 10 token(s) in each box and that they would gain or lose that amount of money according to their selections. They were informed that this was a game of chance and that there was no correlation between the location of the box and the amount of money. They were told to choose one of the two boxes by pressing the “F” or “D” key for the left or right box using the index or middle finger, respectively, of their left hand. There was no time limit for the response. Subsequently, a blank screen was shown for 0 to 2000 ms (*M* = 1000 ms). Participants were told that the presentation of the blank screen indicated that they were waiting for the response from the player. This manipulation allowed the participants to believe they were playing with real persons. Then, the participant’s and the player’s outcomes were presented on the left and right sides of the center of the screen, respectively, for 1500 ms. The number presented signified the amount of money gained or lost. The symbols “+” and “−” to the left side of the number indicated monetary gain and loss, respectively. Participants were then asked to rate how much malicious envy they felt toward the player on a 9-point scale (1 = very low, 9 = very high) by pressing the number on the number keypad of the keyboard using the right hand. Notably, previous studies have shown that the effect of envy on schadenfreude is more evident when envy is malicious than benign (Van de Ven et al., 2015; Lange et al., 2018), we empathized to the participants that the ratings referred to malicious envy rather than benign envy. In addition, research has indicated that benign envy is more likely to occur when inferior persons perceive that they have control to improve their situation, while malicious envy is more likely to occur when inferior persons perceive that the outcome of superior persons is undeserved (e.g., Van de Ven et al., 2012; Lange et al., 2016). The outcomes of box selection were determined by chance, and it was easy for participants to consider the superior outcome of the player as undeserved. Thus, participants were more likely to experience malicious envy than benign envy when they obtained a worse outcome than that of the player. The second round started immediately after the envy assessment. This round was similar to the first round; however, the rating reflected the intensity of pleasure participants felt upon seeing the outcome [ranging from 1 (very low) to 9 (very high)]. Notably, the rating of the degree of pleasure rather than the participant’s feelings of schadenfreude was intended to decrease social desirability issues. The manipulation was in line with that used in previous studies (e.g., Takahashi et al., 2009; Steinbeis and Singer, 2013, 2014; Van de Ven et al., 2015; Baez et al., 2016; Santamaría-García et al., 2017). At the end of the experiment, the participants were asked whether they had participated in similar psychological experiments before and whether they actually believed in the existence of the other players. None of the participants reported that they had experience with similar experiments. All participants reported that they believed they had played with real persons.

**Figure 2:**
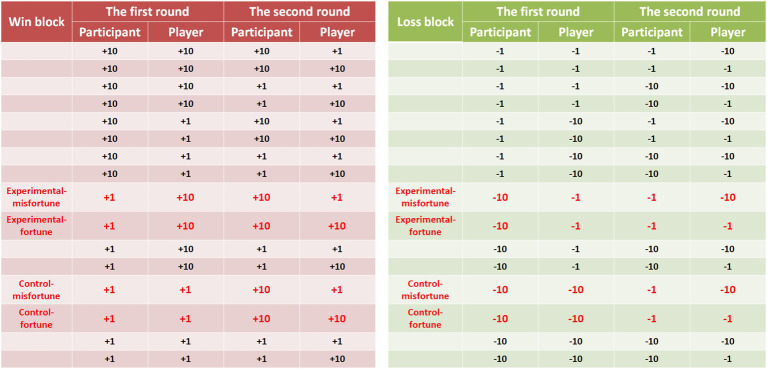
The outcomes between the participants and players for the two rounds of the game in the win and loss block (on the left and right panel, respectively) in Experiment 1. The outcomes in red are those in the experimental and control conditions. The first round of the game is to elicit envy and non-envy emotions, and the second round is to assess the feelings of pleasure (schadenfreude).

The outcomes of the participants and players were in fact predetermined *via* experimental randomization. According to the outcomes for the participants and players in the first and second rounds, there were 16 outcome combinations for each block (see Figure 2 for more details). Based on previous studies (e.g., Dvash et al., 2010; Steinbeis and Singer, 2013, 2014; Lin and Liang, 2021), the outcome combinations written in red were used in the experimental and control conditions of the present study, and the other combinations were used in the filler trials. To elicit malicious envy, the experimental condition in the present study was manipulated such that the outcomes for the first round involved the participants gaining less money than the players in the win block (i.e., participants vs. players = +1 token vs. +10 tokens) or losing more money in the loss block (i.e., −10 tokens vs. −1 token). In the control condition, the outcomes involved both the participants and the players gaining a small amount of money (+1 token vs. +1 token) in the win block or losing a large amount of money (−10 tokens vs. −10 tokens) in the loss block. In the second round, participants in each experimental and control condition encountered two situations to assess schadenfreude. In the first situation (i.e., experimental-misfortune and control-misfortune), the participants gained more money than the players in the win block (i.e., participants vs. players = +10 tokens vs. +1 token) or lost less money in the loss block (i.e., −1 token vs. −10 tokens). In the second situation (i.e., experimental-fortune and control-fortune), both participants and players gained a large amount of money (+10 tokens vs. +10 tokens) in the win block or lost a small amount of money (−1 token vs. −1 token) in the loss block. The reason for using two experimental and control conditions for each block is explained in the “Behavioral Recordings and Analyses” section. Please refer to this section for details.

For each block, there were 20 trials for each experimental and control condition. Each filter trial was presented 4–12 times, for a total of 112 times. In each block, there were 4 breaks. The duration of the break was controlled by the participant. Prior to the actual experiment for each block, there were 16 practice trials to familiarize the participants with the experimental procedure. The experiment (including the practice sessions) lasted approximately 1.5 h.

#### Behavioral Recordings and Analyses

**Figure 3:**
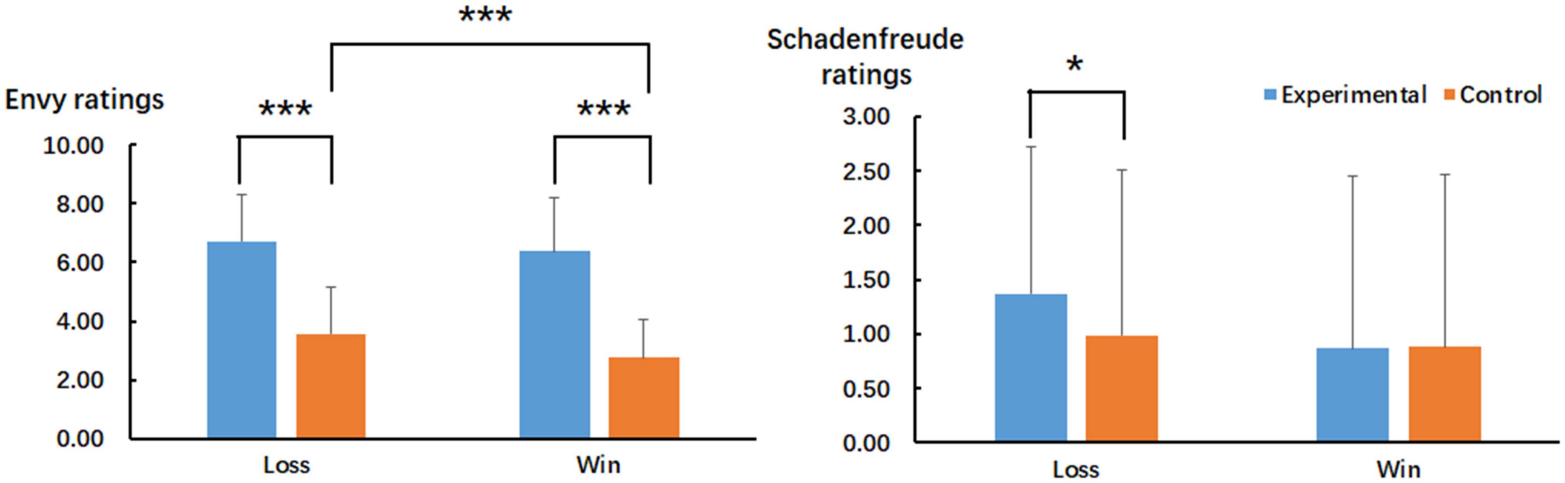
Mean ratings of envy (the left panel) and schadenfreude (the right panel) for the interaction between emotion and block in Experiment 1. Vertical lines indicate the standard deviation of the means. The significance level of the emotional effect is marked by the number of the “^*^” symbol. ^*^p < 0.05 and ^***^p < 0.001, respectively.

Malicious envy and schadenfreude ratings were recorded for each trial. The ratings were then averaged for all trials separately for each emotional category, emotion, and participant. To understand whether malicious envy was elicited successfully, we first averaged the ratings between the experiment-misfortune and experiment-fortune situations and between the control-misfortune and control-fortune situations and then used these averaged ratings to perform 2 × 2 ANOVA with block (win vs. loss) and emotion (experimental vs. control) as within-subject factors. The means and SDs of the ratings are shown in Table 1. Regarding the schadenfreude ratings, participants may feel pleasure when they have a beneficial outcome (e.g., gain 10 tokens rather than 1 token or lose 1 token rather than 10 tokens) irrespective of the outcome of the player. To exclude this effect, we calculated new schadenfreude ratings by subtracting the original schadenfreude ratings in the fortune condition from those in the misfortune condition separately for the experimental and control conditions. This calculation method was based on previous studies (Steinbeis and Singer, 2013, 2014). For these new schadenfreude ratings, we performed a 2 × 2 ANOVA with block (win vs. loss) and emotion (experimental vs. control) as within-subject factors. The means and *SD*s of the ratings are shown in Table 1 and Figure 3.

**Table 1:** Mean ratings of envy and schadenfreude and the SDs for all experimental conditions in Experiment 1.

	Loss		Win		*M*	*SD*	*M*	*SD*
*Envy ratings*				
Experimental	6.72	1.58	6.38	1.84
Control	3.57	1.58	2.75	1.28
*Schadenfreude ratings*				
Experimental (misfortune)	6.50	1.75	6.97	1.45
Experimental (fortune)	5.13	1.83	6.09	1.41
Experimental (misfortune – fortune)	1.37	1.35	0.87	1.58
Control (misfortune)	6.65	1.75	7.16	1.55
Control (fortune)	5.66	1.61	6.28	1.38
Control (misfortune – fortune)	0.99	1.52	0.88	1.59

### Results

#### Envy Ratings

The analyses showed main effects of block [*F*(1, 34) = 28.77, *p* < 0.001,$\;\eta_{p}^{2}$ = 0.46] and emotion [*F*(1, 34) = 142.40, *p* < 0.001,$\;\eta_{p}^{2}$ = 0.81]. The envy ratings were generally higher in the loss condition (*M* ± *SD* = 5.14 ± 1.31) than in the win condition (4.57 ± 1.30) and in the experimental condition (6.56 ± 1.66) than in the control condition (3.16 ± 1.36).

The interaction between the two factors was also significant [*F*(1, 34) = 4.68, *p* = 0.038,$\;\eta_{p}^{2}$ = 0.12]. Bonferroni *post hoc* comparisons showed that the ratings were 3.63 points higher in the experimental condition than in the control condition for the win block (*p* < 0.001, 95% *CI* of the difference = 3.02 to 4.23) and 3.16 points higher for the loss block (*p* < 0.001, 95% *CI* of the difference = 2.57 to 3.74). The ratings in the control condition were 0.81 points higher for the loss block than for the win block (*p* < 0.001, 95% *CI* of the difference = 0.51 to 1.11), whereas the ratings of the win and loss blocks did not significantly differ in the experimental condition (*p* = 0.207).

#### Schadenfreude Ratings

The ANOVA did not show a main effect of block or emotion (*p* ≥ 0.126). There was an interaction between these two factors [*F*(1, 34) = 6.87, *p* = 0.013,$\;\eta_{p}^{2}$ = 0.17]. Bonferroni *post hoc* comparisons showed that schadenfreude ratings were 0.38 points higher in the experimental condition than in the control condition for the loss block (*p* = 0.038, 95% *CI* of the difference = 0.13 to 0.64), whereas the ratings between the experimental and control condition were not significant for the win block (*p* = 1.000). The ratings of the win and loss blocks did not significantly differ in either the experimental or control condition (*p* ≥ 0.400).

### Discussion

In this experiment, we investigated the effect of malicious envy on the degree of schadenfreude separately for gain and loss frames when schadenfreude was explored through social comparisons. The results showed that malicious envy ratings were generally higher in the experimental condition than in the control condition, suggesting that envy was successfully evoked. More importantly, there was an effect of malicious envy on schadenfreude in the loss condition, whereas the effect was not significant in the win condition. The findings might indicate that the effect of malicious envy on schadenfreude occurs only in the context of loss-associated social comparisons.

It is notable that in this experiment, social comparisons associated with malicious envy and schadenfreude were investigated by presenting the exact value of the participants and the player in each round of the game. When participants integrated their outcomes with those of the player for all situations, they were able to compare the overall outcomes between themselves and the player precisely. However, social comparisons are not always precise but sometimes ambiguous. In this case, individuals might be uncertain about their position when all the situations are integrated. Such uncertainty might alter the effect of malicious envy on schadenfreude.

## Experiment 2

In Experiment 2, we aimed to further investigate the effect of malicious envy on schadenfreude in gain and loss frames when social comparisons between the participants and the player were ambiguous. To address this issue, participants in this experiment were not informed about the exact values of the outcomes (e.g., the money gained or lost was +10 RMB or − 10 RMB) and were instead given approximate values (e.g., the money gained or lost was more than or less than 5 RMB). In this case, the participants were uncertain about their position after two rounds (i.e., inferior, equal, or superior). As mentioned in the introduction section, we predict that malicious envy might not increase feelings of schadenfreude irrespective of frame in ambiguous social comparisons.

### Methods

#### Participants

Thirty-four undergraduate students (ranging from 18 to 23 years old, *M* = 20.51, *SD* = 1.01; 23 females) were recruited as participants. The participants did not participate in Experiment 1. The requirements for participant recruitment were the same as those in Experiment 1.

#### Procedure

**Figure 4:**
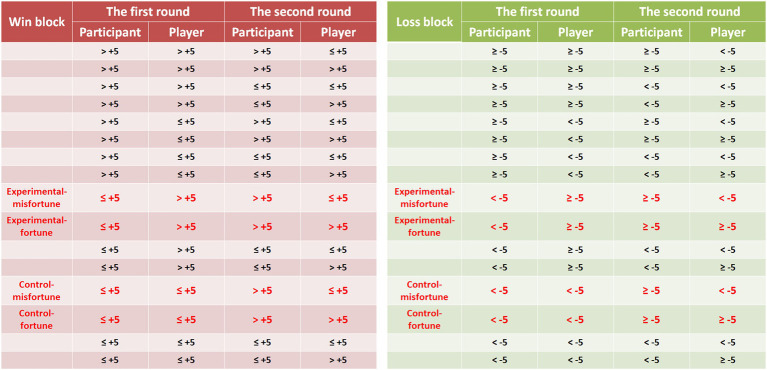
The approximate outcomes between the participants and players for the two rounds of the game in the win and loss block (on the left and right panel, respectively) in Experiment 2. The outcomes in red are those in the experimental and control conditions. The first round of the game is to elicit envy and non-envy emotions, and the second round is to assess the feelings of pleasure (schadenfreude).

As illustrated in Figure 1, the experimental procedure was similar to the procedure in Experiment 1, except for the meaning of the white boxes and the presentation of the outcomes. With respect to the white boxes, participants were told that the amount of money in each white box ranged from 1 to 10 token(s). After the selection of the box, the outcomes for the participants and players were not presented as exact values but as red/green boxes. The color of the boxes indicated the approximate values for the participants and players. For half of the participants, the red box meant that the money gained or lost was more than 5 tokens, and the green box indicated that the money gained or lost was less than or equal to 5 tokens. For the other half, the meaning of the colored boxes was reversed. It was emphasized to the participants that they would not know whether their or the player’s general outcomes were better for the two rounds in a trial. For example, suppose Players A and B choose the red box (representing a gain of more than 5 tokens) in the first round. In the second round, Player A chooses the red box, but Player B chooses the green box (representing no more than 5 tokens). The red boxes for Player A contain 6 tokens in the first and second rounds. For Player B, if the red box in the first round contains 6 tokens and the green box in the second round contains 1 token, then Player A will obtain more money than Player B. However, if the red and green boxes contain 9 tokens and 5 tokens, respectively, Player A will obtain less money than Player B. If the red and green boxes contain 7 tokens and 5 tokens, respectively, Players A and B will gain an equal amount of money. As illustrated, the participants were not told the exact values for themselves or the players in each round and therefore could not determine whose general outcomes were better for the two rounds in the experimental and control conditions. As shown in Figure 4, the outcome combinations written in red were used in the experimental and control trials. The other combinations were used in filler trials.

#### Behavioral Recordings and Analyses

The behavioral recordings and analyses were the same as those in Experiment 1. The means and SDs of the malicious envy ratings and schadenfreude ratings are shown in Table 2.

**Table 2:** Mean ratings of envy and schadenfreude and the SDs for all experimental conditions in Experiment 2.

	Loss		Win		*M*	*SD*	*M*	*SD*
*Envy ratings*				
Experimental	5.45	2.24	5.08	2.12
Control	2.48	1.26	2.06	0.93
*Schadenfreude ratings*				
Experimental (misfortune)	5.26	2.03	6.02	1.74
Experimental (fortune)	4.88	1.63	5.57	1.66
Experimental (misfortune – fortune)	0.38	1.15	0.45	1.75
Control (misfortune)	5.68	1.89	6.49	1.73
Control (fortune)	5.02	1.60	5.81	1.57
Control (misfortune – fortune)	0.66	1.26	0.68	1.86

### Results

#### Envy Ratings

The analyses showed main effects of emotion [*F*(1, 33) = 84.80, *p* < 0.001,$\;\eta_{p}^{2}$ = 0.72] and block [*F*(1, 33) = 9.22, *p* = 0.005,$\;\eta_{p}^{2}$ = 0.22]. The envy ratings were generally higher in the experimental condition (5.26 ± 2.11) than in the control condition (2.27 ± 1.05) and in the loss condition (3.96 ± 1.57) than in the win condition (3.57 ± 1.25).

#### Schadenfreude Ratings

The ANOVA showed a main effect only of emotion [*F*(1, 33) = 5.11, *p* = 0.030,$\;\eta_{p}^{2}$ = 0.13]. The schadenfreude ratings were generally lower in the experimental condition (0.42 ± 1.27) than in the control condition (0.67 ± 1.37). Other main effects or interactions were not significant (*p* ≥ 0.775).

### Discussion

In the present study, we investigated the effect of malicious envy on schadenfreude separately in gain and loss frames when the comparisons between the participants and the player were ambiguous. Similar to the results in Experiment 1, the results in Experiment 2 showed that envy ratings were generally higher in the experimental condition than in the control condition, suggesting successful elicitation of malicious envy. Different from Experiment 1 and previous studies (e.g., Feather and Sherman, 2002; Feather and Nairn, 2005; Van Dijk et al., 2006; Takahashi et al., 2009; Feather et al., 2013; Van de Ven et al., 2015; Baez et al., 2016, 2018; Santamaría-García et al., 2017; Lin and Liang, 2021), however, we observed lower schadenfreude ratings in the experimental condition than in the control condition in both gain and loss frames. The findings suggest that malicious envy reduces the degree of schadenfreude irrespective of frame when social comparisons are ambiguous.

## General Discussion

The present study investigated whether malicious envy influenced schadenfreude in different frames when both malicious envy and schadenfreude were explored through social comparisons. The results showed that when the outcomes of the participant and the player were precise, malicious envy increased the degree of schadenfreude in the loss frame, whereas this was not the case in the gain frame. In the case of ambiguous outcomes, however, schadenfreude was reduced by malicious envy irrespective of frame. In general, the findings might suggest that malicious envy does not always increase schadenfreude particularly when malicious envy and schadenfreude are elicited by social comparisons.

When individuals know about the exact outcomes between themselves and others in the loss frame, increased schadenfreude by malicious envy might be explained by the achievement of the motivational goal of malicious envy and/or reduced psychological pain. The motivation goal of malicious envy is to damage the position of the superior other. When misfortune befalls the other, the superior position of the other might be weakened. In this case, the motivational goal might be more or less achieved, resulting in positive feelings (i.e., schadenfreude; Van de Ven et al., 2015). Accordingly, in the present study, when participants lost more money than the player in the first round of the game, they may have experienced malicious envy and have been motivated to pull the player down. In the subsequent round, this motivation goal was achieved when a misfortune outcome occurred to the player, resulting in increased feelings of schadenfreude.

In terms of psychological pain, envy is thought to be an emotion related to pain (e.g., Lange et al., 2018). Other’s misfortune that can relieve social pain is regarded as a reward and, thus, activates reward-related striatal regions. This increased activation leads to increased degree of schadenfreude (Takahashi et al., 2009). For the present study, participants might feel painful when they obtained a worse outcome than the player in the first round of the game. In the subsequent round, the player’s misfortune might reduce the painful feeling elicited in the preceding round, leading to increased schadenfreude.

However, the effect of malicious envy on schadenfreude was not significant in the gain frame. After all the situations are presented (e.g., Round 1 and 2), individuals might calculate the general outcomes for themselves and others and to compare their general outcomes with those of the others. The comparison in the present study might have participants affirm that their position was no longer inferior but was similar to the position of the player, which might reduce schadenfreude (Van Dijk et al., 2011). More importantly, it has been shown that individuals in the gain frame focus more on the differences between their own and the other player’s outcomes than for those in the loss frame (De Dreu et al., 1992, 1994; Poppe and Valkenberg, 2003). Therefore, general outcome comparisons and self-affirmation of the increased position might be more likely to occur in the gain frame, resulting that reduced feelings of schadenfreude are more probable to appear in this frame.

The differential effects of malicious envy on schadenfreude in gain and loss frames might also be explained by loss aversion. The prospect theory proposes that subjective experiences of monetary loss are more evaluable than the experiences of gains (Tversky and Kahneman, 1979). For the present study, participants in both gain and loss frames might realize their position was similar to the position of the player after calculating the general outcomes for all the situations (e.g., Round 1 and 2). Due to loss aversion, however, the evaluations to the similar position might be different in gain and loss frames. For example, participants in the loss frame might be likely to evaluate the similar position as an unfavorable outcome, whereas this might be not the case for participants in the gain frame. The specific evaluation pattern in the loss frame might lead to the maintenance of schadenfreude.

Surprisingly, when participants were told about ambiguous outcomes for themselves and the player, the present study showed a reversed effect of malicious envy on schadenfreude. Similar to precise social comparisons, participants might integrate their own outcomes and the outcomes of the player for all situations and affirm that the participants’ position was not as inferior as it was used to be. This issue might have reduced the feelings of schadenfreude (Van Dijk et al., 2011) and, thus, the effect of malicious envy on schadenfreude (particularly in the gain frame). More importantly, due to ambiguous outcomes, participants were uncertain whether their position was still inferior or not, even though a misfortune occurred to the player. It has been reported that uncertainty reduces pleasantness feelings evoked by positive stimuli (Lin et al., 2012, 2015, 2020). Thus, in the present study, schadenfreude to the player’s misfortune might be generally reduced by outcome uncertainty. Moreover, in the experimental condition, the outcomes between the participants and the player were reversed for the two rounds, and thus, the relative position between them might be considered to be quite uncertain. Even though the relative position was also uncertain in the control condition, the outcomes were similar between participants and the players in the first round but better for the participants than for the player in the second round, which looked that the overall outcomes were better for the participants. That is, participants might feel more uncertain in the experimental condition than in the control condition, as they in the control condition were more likely to think of obtaining a superior position, even though the exact probability of obtaining such a position in the present study was similar between the experimental and control conditions (48 and 49%, respectively). Therefore, schadenfreude might be reduced to a larger extent for the experimental condition than for the control condition, which leads to a reversed effect of malicious envy on schadenfreude irrespective of frame.

The current findings might be in line with previous studies, which indicated that envy increases feelings of schadenfreude only under certain circumstances. For instance, the effect occurs when envy is malicious but not when it is benign (Van de Ven et al., 2015; Lange et al., 2018), when enviable persons are competitive out-group but not when they are non-competitive or in-group members (Cikara and Fiske, 2013), and when enviable and envious persons have the same sex but not when they have different sexes (Van Dijk et al., 2006). The findings in the present study further revealed that the effect of malicious envy on schadenfreude was observed only when the scenarios involved loss-associated and precise social comparisons. Otherwise, the effect of malicious envy on schadenfreude was reduced or even reversed.

However, the findings in the present study (except when participants knew the exact outcomes for themselves and the players in the loss frame) are inconsistent with those of previous studies (e.g., Van Dijk et al., 2006; Takahashi et al., 2009; Cikara and Fiske, 2013; Feather et al., 2013; Baez et al., 2016, 2018; Santamaría-García et al., 2017; Lin and Liang, 2021), which showed that envy increased feelings of schadenfreude. Both our present study and previous studies used abovementioned approaches that enlarged the effect of envy on increased schadenfreude (i.e., eliciting malicious envy and presenting competitive out-group and similar enviable persons). Different from our present study, however, the scenarios regarding schadenfreude in previous studies did not involve social comparisons (e.g., participants were told a story in which the person met with misfortune, were asked to imagine a misfortune befalling the person or saw a player whom they were playing with obtained a bad outcome). Individuals could not integrate the outcomes of social comparisons for all situations. In this case, individuals might be likely to believe that they are still in an inferior position even though social positions of others are pulled down by the misfortune. In the current study, however, not only malicious envy but also schadenfreude was investigated in the context of social comparisons, and outcomes for social comparisons could be integrated to some extents. When a misfortune occurred to the others, individuals know that their social position is not as inferior as the position that was used to be. This knowledge might alter the effect of malicious envy on schadenfreude particularly when social comparisons are ambiguous and/or gain-associated.

In general, the findings obtained in this study appear to be in line with theories regarding envy (e.g., the malicious envy theory, the dual envy theory, and the pain-driven dual envy theory), supporting the effect of envy, particularly malicious envy, on schadenfreude irrespective of frame (e.g., Van Dijk et al., 2006; Takahashi et al., 2009; Cikara and Fiske, 2013; Feather et al., 2013; Van de Ven et al., 2015; Baez et al., 2016, 2018; Santamaría-García et al., 2017; Lange et al., 2018; Lin and Liang, 2021). The findings in the present study extend previous theories by showing that the effect of malicious envy on schadenfreude occurs even when schadenfreude is assessed in the context of social comparisons. However, the findings provide new insights in that the mechanisms underlying the effect of malicious envy on schadenfreude might be more complicated than the mechanisms described by the theories. Specifically, when social comparisons associated with malicious envy and schadenfreude are precise, malicious envy increases schadenfreude in the loss frame but not in the gain frame. Moreover, the effect of malicious envy on schadenfreude is reversed irrespective of frame when social comparisons are ambiguous. The current study might contribute to further understanding of how malicious envy influences degree of schadenfreude.

In addition, recent evidence pointed out that malicious social-moral emotion resulted in the appearance and/or maintenance of pathological behaviors (e.g., dishonest, criminal, and antisocial behaviors; e.g., Panasiti and Ponsi, 2017; Franco-O’Byrne et al., 2021). Consistent with the evidence, our findings (at least at loss-associated precise social comparisons) and several other studies (e.g., Van Dijk et al., 2006; Takahashi et al., 2009; Cikara and Fiske, 2013; Feather et al., 2013; Baez et al., 2016, 2018; Santamaría-García et al., 2017) also showed that malicious social emotions (e.g., malicious envy) increased other pathological emotions (e.g., schadenfreude). It has been suggested that whether pathological behaviors and emotions are elicited involve a conflict between extrinsic (e.g., self-related benefits) and intrinsic goals (e.g., social norms; Mazar et al., 2008). Once extrinsic goals are more salient than intrinsic goals, pathological behaviors, and emotions might occur (Panasiti and Ponsi, 2017). For example, other’s misfortune elicits either empathy or schadenfreude. The salient extrinsic goal of pulling others down (i.e., the motivational goal of malicious envy) increases schadenfreude and reduces empathy. Therefore, we speculate that social emotions involving extrinsic goals might be an important factor in eliciting pathological behaviors and emotions. This might also explain why individuals who have experiences in extremely pathological behaviors might have abnormal processes in social emotions and relationships (e.g., Jankowski and Takahashi, 2014), for example, offenders experienced reduced feelings of envy and schadenfreude (Franco-O’Byrne et al., 2021).

Finally, we would like to note some of the limitations of the present study and suggest future directions. First, to further understand the effect of malicious envy on schadenfreude, future studies could investigate the correlation/regression between malicious envy and schadenfreude ratings. In the present study, envy ratings were calculated by averaging the original ratings in the misfortune and fortune conditions, whereas schadenfreude ratings were calculated by subtracting the original ratings in the fortune condition from the ratings in the misfortune condition. The difference in the calculation approaches may affect the correlation/regression between these two ratings. Future studies might consider a single calculation approach that can be used for both ratings to further investigate their relation. Second, previous studies in which schadenfreude were not investigated in the context of social comparisons have suggested that schadenfreude is affected more by malicious envy than benign envy (e.g., Van de Ven et al., 2015; Lange et al., 2018). However, it remains unclear whether this is also the case when schadenfreude is investigated in the context of social comparisons. Future studies might investigate this issue for more details. Finally, the social comparison task in the present study required the participants to complete numerous trials, which may have led to carry-over effects or fatigue effects. Future studies should identify ways to reduce the number of trials while preserving high reliability and validity.

## Conclusion

The present study showed that malicious envy increased feelings of schadenfreude occurred in the loss frame but not in the gain frame, when participants knew the exact outcomes of themselves and the players in each trial. Without this knowledge, however, there was a reversed effect of malicious envy on schadenfreude irrespective of frame. The findings of the present study highlight the effect of malicious envy on schadenfreude when both envy and schadenfreude were elicited through social comparisons.

## Data Availability Statement

The raw data supporting the conclusions of this article will be made available by the authors, without undue reservation.

## Ethics Statement

The studies involving human participants were reviewed and approved by the academic committee of School of Public Administration, Guangdong University of Finance. The patients/participants provided their written informed consent to participate in this study.

## Author Contributions

HL contributed to conception and design of the study and wrote the first draft of the manuscript. HL and JL performed the data collection and statistical analysis. All authors contributed to manuscript revision, read, and approved the submitted version.

## Funding

This work was supported by the National Natural Science Foundation of China under Grant (No. 31800940).

## Conflict of Interest

The authors declare that the research was conducted in the absence of any commercial or financial relationships that could be construed as a potential conflict of interest.

## Publisher’s Note

All claims expressed in this article are solely those of the authors and do not necessarily represent those of their affiliated organizations, or those of the publisher, the editors and the reviewers. Any product that may be evaluated in this article, or claim that may be made by its manufacturer, is not guaranteed or endorsed by the publisher.
